# Effect of Anodal Direct-Current Stimulation on Cortical Hemodynamic Responses With Laser-Speckle Contrast Imaging

**DOI:** 10.3389/fnins.2018.00503

**Published:** 2018-07-26

**Authors:** Shuo Hu, Tao Zheng, Yanchao Dong, Juan Du, Lanxiang Liu

**Affiliations:** ^1^Institute of Electrical Engineering, Yanshan University, Qinhuangdao, China; ^2^Department of Magnetic Resonance Imaging, Qinhuangdao Municipal No. 1 Hospital, Qinhuangdao, China

**Keywords:** direct-current stimulation, laser-speckle contrast imaging, cerebral blood flow, hemodynamic response, rat

## Abstract

Transcranial direct-current stimulation (DCS) offers a method for noninvasive neuromodulation usable in basic and clinical human neuroscience. Laser-speckle contrast imaging (LSCI), a powerful, low-cost method for obtaining images of dynamic systems, can detect regional blood-flow distributions with high spatial and temporal resolutions. Here, we used LSCI for measuring DCS-induced cerebral blood flow in real-time. Results showed that the change-rate of cerebral blood flow could reach approximately 10.1 ± 5.1% by DCS, indicating that DCS can increase cerebral blood flow and alter cortical hemodynamic responses. Thus, DCS shows potential for the clinical treatment and rehabilitation of ischemic strokes.

## Introduction

Transcranial direct-current stimulation (DCS), which utilizes weak currents to regulate the activity of neurons in the cerebral cortex, is a noninvasive method for performing transcranial stimulation. In DCS, electrodes are placed on the scalp, and a weak direct current is applied to the cerebral cortex, changing its excitability (Ferrucci and Priori, 2014). The effects of DCS have the characteristics of polarization. Anodal stimulation can enhance cortical excitability, and cathodal stimulation can reduce cortical excitability. Stimulation effects are determined by measuring the duration and current intensity of the stimuli. If the stimulus duration is long enough, changes in cortical excitability can last up to 1 h. The effect can be controlled by adjusting the current intensity and duration of the stimuli (Antal et al., 2003; Siebner et al., 2004; Jeffery et al., 2007; Ragert et al., 2008; Hunter et al., 2009; Tanaka et al., 2009). The duration of continuous stimulation may play an important role in prolonging the duration of DCS (Kwon et al., 2008). Clinical applications of DCS have been proven useful in treating many conditions, such as fibromyalgia (Marlow et al., 2013), spinal-cord injuries (Soler et al., 2010), schizophrenia (Agarwal et al., 2013), depression (Nitsche et al., 2009), Parkinson's disease (Benninger et al., 2010), epilepsy (San-juan et al., 2015), and strokes (Baker et al., 2010). Previous studies also demonstrated that DCS could alter blood flow in the brain (Paquette et al., 2011; Zheng et al., 2011). Researchers using either laser Doppler flowmetry (LDF) (Wachter et al., 2011), functional near-infrared spectroscopy (Giovannella et al., 2018) or functional magnetic resonance imaging (fMRI) (Baudewig et al., 2001) for measuring cerebral blood flow observed that blood flow changed across a given region. Thus, the techniques could not indicate the structure of the blood vessels or determine the changes of blood-flow velocity in the blood vessels. This problem can be solved by using laser-speckle contrast imaging (LSCI) with DCS to study hemodynamics.

Laser-speckle contrast imaging (LSCI) technology, proposed by Briers and Webster (1996) and Briers et al. (1999), can obtain regional blood-flow distributions without scanning. In addition, this method uses a reflection model to measure blood flow and does not require the blood vessels to be transparent. In addition to its capability to image rapid blood flow (Zakharov et al., 2009; Dunn, 2012), LSCI also offers many advantages over other traditional methods, such as high spatial and temporal resolutions, imaging without contrast agents and real-time imaging. LSCI has seen wide use in nerve blood-flow imaging and is especially suited to the study of neural activity and hemodynamics (Ponticorvo and Dunn, 2010; Li et al., 2011, 2014; Zhang et al., 2012; Chao and Li, 2016).

In our study, we built up an LSCI system for real-time monitoring of rat cerebral hemodynamics induced by DCS. The laser-speckle contrast images obtained before and after DCS were recorded, and the distribution of blood flow was reconstructed by employing temporal laser-speckle contrast imaging methods. The rate of change of cerebral blood flow (ΔCBF) was determined to perform a quantitative evaluation of changes in cerebral blood flow.

## Materials and methods

### Experimental setup for LSCI and DCS

**Figure 1:**
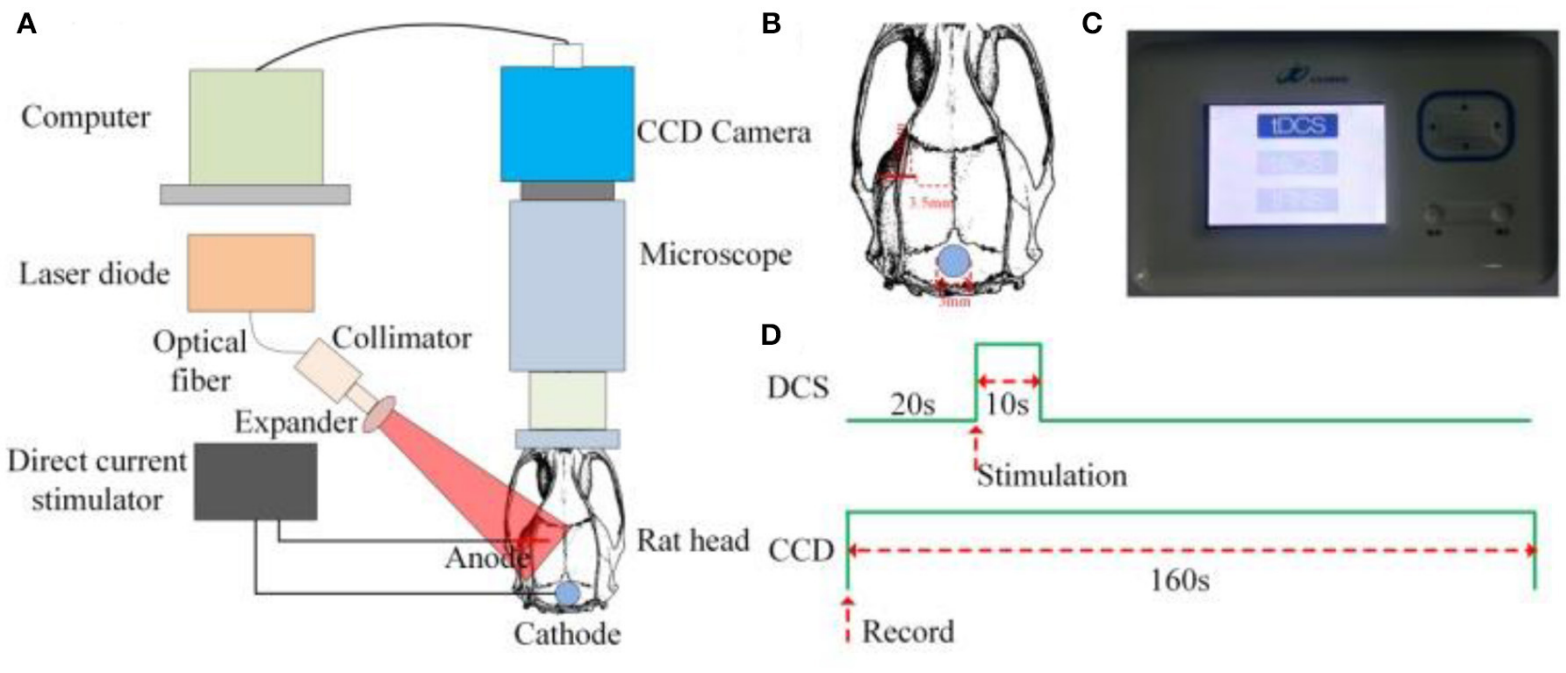
Experimental setup for LSCI and DCS **(A)** Schematic of the experimental setup. **(B)** AP and ML coordinates of position, 3.5 and 2.5 mm, respectively, of the anode-stimulating electrode. **(C)** Photograph of the direct-current stimulator. **(D)** Sequential diagram of the experiment.

The schematic of the experimental setup is shown in Figure 1A. A diode laser (MW-SGX-635; 635 nm, 20 mW; Leishi Co., Ltd., Changchun City, China) coupled to a 600-μm-diameter silica optical fiber was used as the light source. The light from the optical fiber illuminated the brain tissue. The light scattered by the brain tissues and blood vessels passed through a trinocular stereo microscope (XTL-165 Series, Phenix Optical Holding Stock Co., Ltd., Shangrao City, China) and was recorded by a charge-coupled device (CCD) camera (pco.pixelfly usb, 14 bit, PCO AG, Kelheim, Germany) that was mounted to the trinocular stereo microscope. The laser-speckle images were acquired at 21 fps with an exposure time of 20 ms. Figure 1D presents a sequential diagram of the experimental procedure. In the experiment, the CCD camera recorded images for 160 s, including periods of 20 s before initiating an application of DCS to establish a baseline, 10 s of DCS, and 130 s after the DCS.

### Animal surgery and anesthesia

A total of 11 Sprague-Dawley rats (3-month-old males, body masses of approximately 270 g) were selected for experiment. The study was approved by the ethics committee of Qinhuangdao First Hospital. Surgical anesthesia was induced with sodium pentobarbital (3%, 5 mg/100 g, i.p.). The anesthetized rats were fixed to a stereotaxic apparatus (ST-5ND-C, Stoelting Co., Wood Dale, U.S.A.) with ear bars and a clamping device and body temperature was maintained at 37°C using a heating pad (NF, Nuanfeng Co., Guangdong, China). The fur covering each rat's skull was shaved, and the skin was cleaned with a 0.9% sodium chloride physiological solution. Last, the iodine was used to disinfect for the skin. The scalp was cut along the midline of the skull, and the subcutaneous tissue and periosteum were removed. A section of the skull was removed to expose the brain tissue in an area of approximately 0.5 × 0.5 cm.

### Temporal laser-speckle contrast imaging

After completion of the surgical procedures, a customized anode-stimulating electrode with a diameter of 150 μm was mounted on the surface of the cerebral cortex. The anteroposterior (AP) and mediolateral (ML) coordinates of the position of the anode-stimulating electrode were 3.5 and 2.5 mm, respectively, as shown in Figure 1B. A customized circular cathode-stimulating electrode with a diameter of 3 mm was fixed on the skull after lambda. A direct-current stimulator (Jielian Technology Co., Ltd., Yiwu City, China), shown in Figure 1C, supplied direct current of 15 μA.

Na Li et al. presented a method for calculating blood flow with temporal LSCI (Li et al., 2009). The velocity information in the blur can be extracted and mapped to contrast using statistical arguments. Thus, the laser-speckle contrast CM can be defined by

(1) $$\begin{array}{l} C_{M}=\frac{\langle I_{M}^{2}\rangle -{\langle I_{M}\rangle }^{2}}{{\langle I_{M}\rangle }^{2}}=\frac{\sigma_{M}^{2}}{{\langle I_{M}\rangle }^{2}}. \end{array}$$

where 〈*I*_*M*_〉 and $\langle I_{M}^{2}\rangle$ are the average and the mean-square values of the time-varying speckle intensity over M observations, respectively, and $\sigma_{M}^{2}$ is the square of the standard deviation of the time-varying speckle intensity. The contrast CM can be calculated over time using a time stack of images. In this case, a pixel window is moved across a time stack of M images to obtain the statistics leading to a temporally contrasted image.

The velocity v of the scatting particles and the speckle contrast CM are related by the integration time, where

(2) $$\begin{array}{l} v=\frac{2w}{T}\frac{{\langle I_{M}\rangle }^{2}}{\langle I_{M}^{2}\rangle -{\langle I_{M}\rangle }^{2}}=\frac{2w}{T}\frac{1}{C_{M}}. \end{array}$$

Here, T is the integration time and *w* is the radius of the illuminating beam.

In our trials, we obtained one contrast image from 63 original laser-speckle images. Because the laser-speckle images were acquired at 21 fps, the time resolution of the laser-speckle contrast imaging was 3 s. We determined the ΔCBF to evaluate quantitatively the change of cerebral blood flow, where

(3) $$\begin{array}{l} \Delta CBF=\frac{{\left(1/C_{M}\right)}_{Exp}-{\left(1/C_{M}\right)}_{Bas}}{{\left(1/C_{M}\right)}_{Bas}}\times 100\%. \end{array}$$

Here, (*1/C*_*M*_) _Exp_ is the reciprocal of the laser-speckle contrast with FUS, and (*1/C*_*M*_)_*Bas*_ is the reciprocal of the laser-speckle contrast before DCS.

## Results

**Figure 2:**
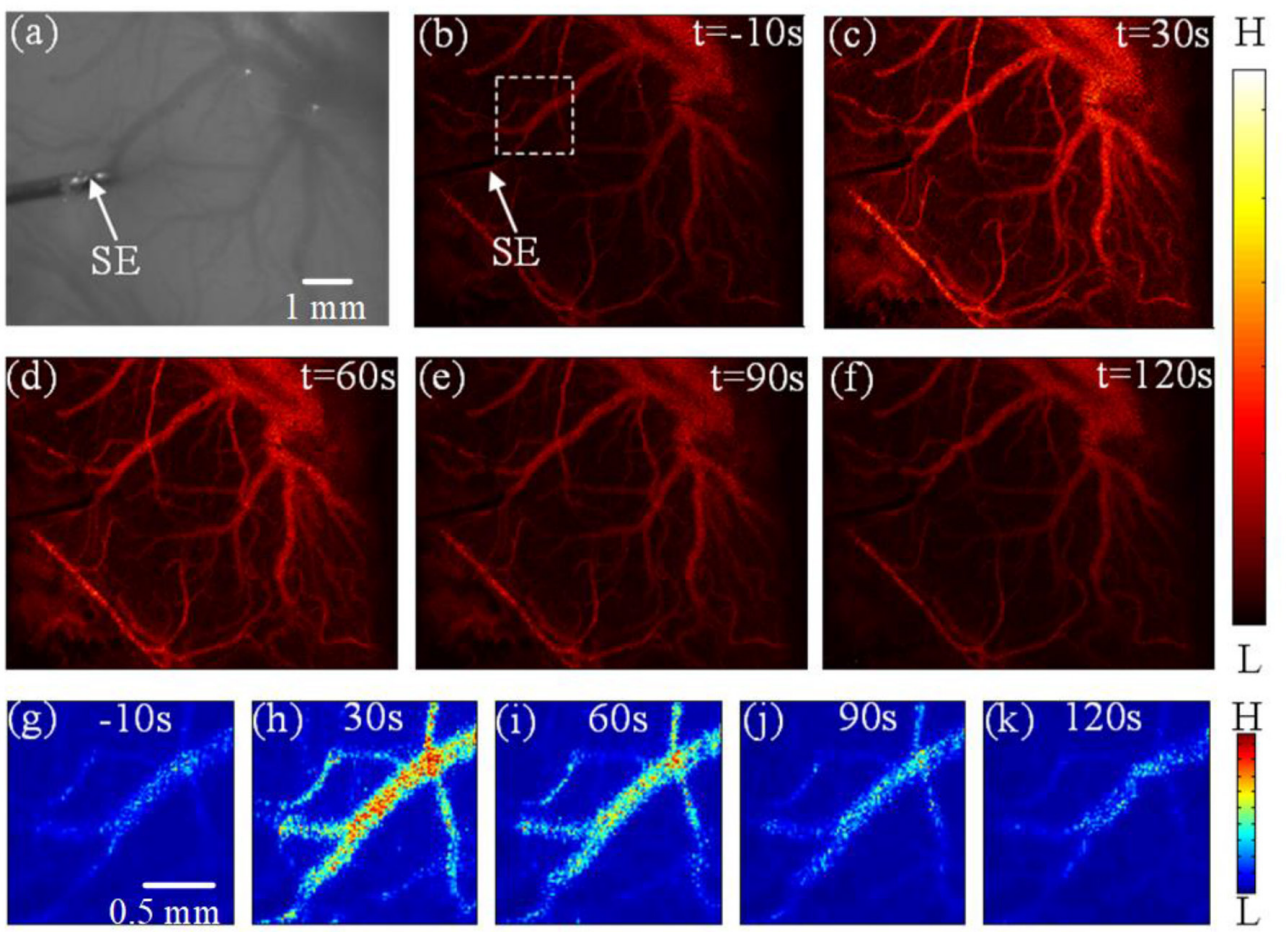
Experimental results depicting the effects of DCS **(a)** Photograph of the imaging region. **(b–f)** Laser-speckle contrast images of blood flow in the rat cerebral cortex at times of −10 s, 30 s, 60 s, 90 s, and 120 s, respectively. **(g–k)** the enlarged image of interested region marked by white dashed frame in **(b)** at −10, 30, 60, 90, and 120 s, respectively. Here, SE notes the stimulating electrode.

Figure 2 shows the laser-speckle contrast images of one rat before and after DCS. Compared with the results at −10 s, shown in Figure 2a, the blood-flow velocity significantly accelerated at 30 s, seen in Figure 2b. The blood-flow velocity gradually decreased during the period from 30 to 90 s, as shown in Figures 2c,d. From 90 to 120 s, as shown in Figures 2d,e, the blood-flow velocity gradually grew stable and approximated the results obtained before stimulation at −10 s. In order to make the difference obvious, we choose and zoom in an interested region marked by white dashed frame in Figure 2b. And then we obtain the enlarged image shown in Figures 2g–k at −10, 30, 60, 90, and 120 s, respectively. We also can clearly see that the blood-flow velocity significantly accelerated at 30 s, and gradually decreased during the period from 30 to 90 s. These results show that DCS can affect the blood-flow velocity. The change of blood-flow velocity traversed three stages: enhancement, weakening, and recovery of stability.

**Figure 3:**
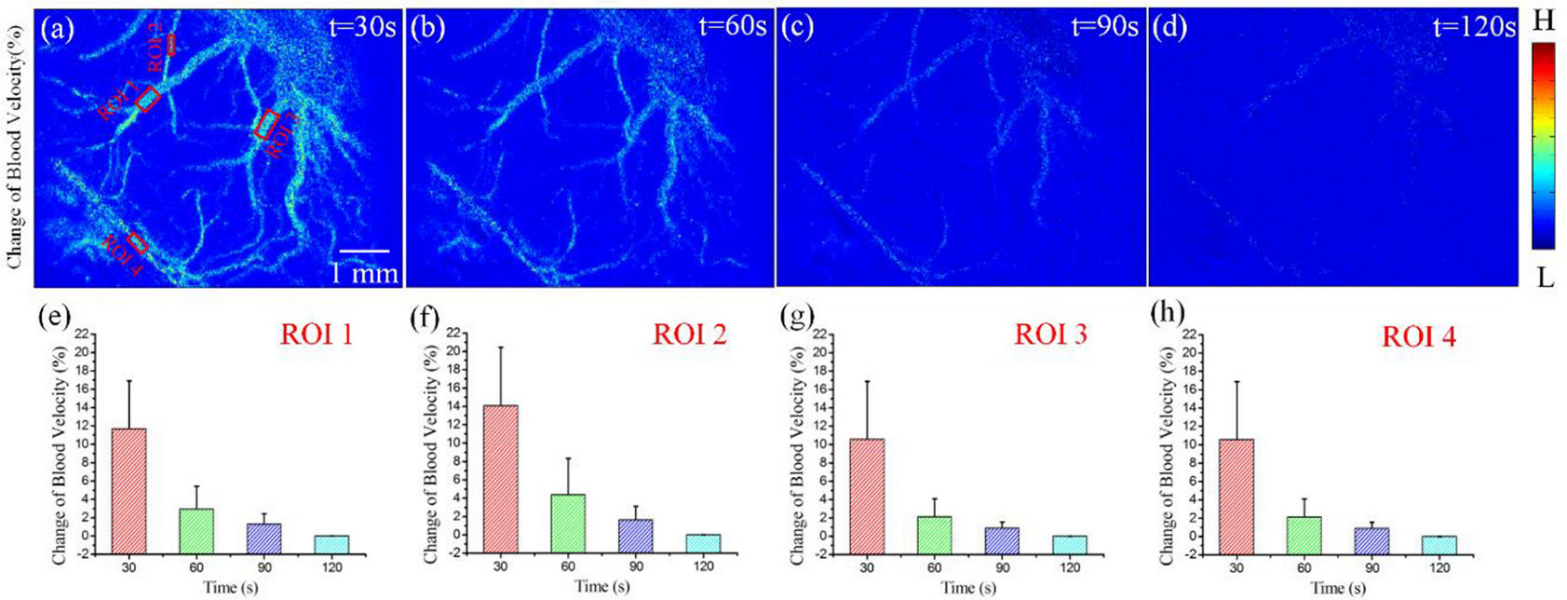
ΔCBF for varying periods of DCS. **(a–d)** ΔCBF with DCS at times 30, 60, 90, and 120 s, respectively. **(e–h)** ΔCBF at different times for ROI1, ROI2, ROI3, and ROI4, respectively.

We calculated the ΔCBF for three regions of interest (ROI) to evaluate quantitatively the effects of DCS on cerebral blood flow. Figures 3a–d depict the results of ΔCBF at 30, 60, 90, and 120 s, respectively. We calculated the ΔCBF, obtaining the average values of all pixels in ROI1, ROI2, ROI3, and ROI4; the corresponding results appear in Figures 3e–h, respectively. Figure 3e presents the ΔCBF of ROI1 at 30, 60, 90, and 120 s as 11.7 ± 5.2%, 2.9 ± 2.5%, 1.3 ± 1.1%, and 0.8 ± 0.68%, respectively, where each result is of the form of mean ± s.t.d. Figure 3f shows the ΔCBF of ROI2 at 30, 60, 90, and 120 s as 14.1 ± 6.3%, 4.4 ± 3.8%, 1. 6± 1.5%, and 0.7 ± 0.59%, respectively. Then, Figure 3g depicts the ΔCBF of ROI3 at 30, 60, 90, and 120 s as 11.7 ± 5.6%, 2.3 ±2.1%, 1.0 ± 0.8%, and 0.7 ± 0.65%, respectively. Finally, Figure 3h presents the ΔCBF of ROI4 at 30, 60, 90, and 120 s as 10.6 ± 6.3%, 2.2 ± 1.9%, 0.9 ± 0.6%, and 0.6 ± 0.53%, respectively. Thus, these results show that DCS significantly increased the blood flow in the rat cerebral cortex.

**Figure 4:**
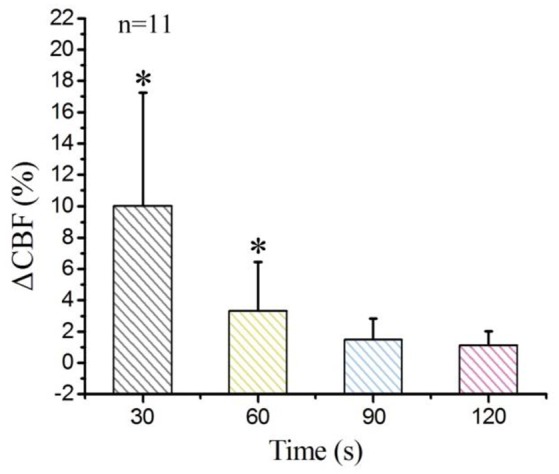
Statistical analysis of ΔCBF at different times for the 11 examined rats. *P < 0.05, paired *t*-test.

Finally, we calculated the average values of all pixels in the ROIs of each rat, and then obtained the average values across 11 rats. The statistical analysis results appear in Figure 4. The laser-speckle contrast at −10 s served as the baseline. Then, we calculated the ΔCBF at 30, 60, 90, and 120 s and determined that the corresponding values were 10.1 ± 5.1%, 3.3 ± 2.1%, 1.5 ± 1.1%, and 1.1 ± 0.89%, respectively. Here, the values have the form of mean ± s.t.d. and were determined by conducting a paired *t*-test with a baseline at every imaging time point, where ^*^P < 0.05.

## Discussion

First, we qualitatively analyzed the laser-speckle contrast image obtained from one rat. The results showed that DCS enhanced cerebral blood flow. At first, the blood-flow velocity increased with DCS, and then, as time passed, it decreased until remaining steady at 90 s. Next, we used the ΔCBF to analyse quantitatively the changes of blood flow for four ROIs. The results showed that the blood flows of three regions all increased; the values of ΔCBF of ROI1, ROI2, ROI3, and ROI4 were 11.7 ± 5.2%, 14.1 ± 6.3%, 11.7 ± 5.6%, and 10.6 ± 6.3%, respectively, at the time of 30 s. These results indicate that DCS significantly increases the cerebral blood flow. We conclude that the direct-current-induced nerve excitement and increased oxygen consumption led to the increases of blood-flow velocity. Finally, we analyzed the ΔCBF for the 11 rats and the statistical analysis of the results also showed that the ΔCBF increased with DCS.

Nan Li et al. studied hemodynamic responses produced by pulsed electric stimulation with a 1 ms pulse train at 5 Hz for a duration of 10 s using a stimulator (Li et al., 2009). They found that the pulsed electric stimulation can enhance the cerebral blood flow of rat. In our paper, we evaluate the blood flow with DCS. Compared to pulsed electric stimulation, DCS is a noninvasive neuromodulation technology. The different results were reduced by different simulation model and parameters.

A previous study utilized LDF to evaluate cerebral hemodynamic responses induced by DCS (Wachter et al., 2011). However, in our study, we used LSCI method to measure the blood flow induced by DCS. First, in imaging technology, LDF that is a method for evaluating cerebral hemodynamic responses can only provide information about the cerebral blood flow in the local region of the brain (approximately 1 mm^3^), and the spatial resolution is not high enough (Shepherd and Berg, 1990). Compared to LDF, LSCI can only not obtain regional blood-flow distributions without scanning, but also offers many advantages such as high spatial and temporal resolutions, imaging without contrast agents and real-time imaging. LSCI can measure the spread of cerebral blood flow change in horizontal direction due to two-dimensional imaging. However, since the LSCI can not supply depth information of the imaging region, we do not obtain the spread of cerebral blood flow change in depth direction. A major contribution on understanding of mechanism of DCS is that we measured the spread of cerebral blood flow change in horizontal direction and monitored the cerebral blood flow change of each blood vessel as shown in Figure 3a. Second, in imaging results, the previous study showed that anodal DCS can increase cerebral blood flow for periods up to 30 min. In our study, the duration of ΔCBF induced by DCS was not more than 90 s, significant less than the duration employed in the previous study. In that study, the current intensity and duration of the stimulus were 100 μA and 15 min, respectively. In our study, in contrast, the current intensity and duration of the stimulus were 15 μA and 10 s, respectively. We conclude that low stimulus-current intensity and short stimulus duration reduce the duration of ΔCBF. In further research, we will stimulate the brain cortices of rats with different current intensities and stimulus durations to analyze the relationship between duration of ΔCBF and stimulus current intensity.

In this study, we merely placed the anode electrode against the surface of the brain for direct electrical stimulation and did not monitor the blood flow under DCS. Results from this work will form the foundation for further studies in which we will use LSCI to monitor changes of blood flow with DCS.

Direct-current stimulation (DCS) can affect the resting potential of the neuronal membrane and neural activity (Agarwal et al., 2013; Zhang and Song, 2018). Anodal DCS has excitatory effects, and cathodal DCS has inhibitory effects on the underlying cortex. As we know, neurons and astrocytes directly regulate the local blood flow within the capillaries, resulting in local neurovascular coupling (Liao et al., 2013; Kalchenko et al., 2014). Therefore, DCS can causes the change of hemodynamics by neurovascular coupling.

Thus, anodal DCS can enhance cerebral blood flow and alter cortical hemodynamic responses. This finding indicates that DCS shows potential for use as a powerful and noninvasive method for creating therapeutic interventions with ischemic strokes.

## Ethics statement

All procedures were carried out in accordance with the Animal Ethics and Administrative Council of Yanshan University and Hebei Province, People's Republic of China. Animal owners agreed that the rats were used in the experiment. There is not any additional considerations.

## Author contributions

SH, TZ, and LL designed and coordinated the study, SH, TZ, JD, and LL carried out experiment and data process, and drafted the manuscript. All authors gave final approval for publication.

### Conflict of interest statement

The authors declare that the research was conducted in the absence of any commercial or financial relationships that could be construed as a potential conflict of interest.
